# A small molecule NRF2 activator BC-1901S ameliorates inflammation through DCAF1/NRF2 axis

**DOI:** 10.1016/j.redox.2020.101485

**Published:** 2020-03-04

**Authors:** Yanwen Chen, John W. Evankovich, Travis B. Lear, Ferhan Tuncer, Jason R. Kennerdell, Daniel P. Camarco, Morgan S. Shishido, Yuan Liu, Bill B. Chen

**Affiliations:** a Aging Institute, University of Pittsburgh, Pittsburgh, PA, 15213, USA; b Department of Gastroenterology, Ruijin Hospital, Shanghai Jiaotong University School of Medicine, Shanghai, 200025, China; c Department of Medicine, Acute Lung Injury Center of Excellence, University of Pittsburgh, Pittsburgh, PA, 15213, USA; d Department of Environmental and Occupational Health, School of Public Health, University of Pittsburgh, Pittsburgh, PA, 15261, USA; e McGowan Institute for Regenerative Medicine, University of Pittsburgh, Pittsburgh, PA, USA; f Vascular Medicine Institute, University of Pittsburgh, Pittsburgh, PA, 15213, USA

**Keywords:** NRF2, DCAF1, High throughput screening, Drug discovery, Ubiquitination, E3 ligase

## Abstract

NRF2 is a master regulator of cellular anti-oxidant and anti-inflammatory responses, and strategies to augment NRF2-dependent responses may beneficial in many diseases. Basal NRF2 protein level is constrained by constitutive KEAP1-mediated degradation, but in the presence of electrophiles, NRF2 ubiquitination is inhibited. Impeded NRF2 degradation increases NRF2 protein, resulting in up-regulation of anti-oxidant gene transcription, and decreased inflammation. KEAP1-independent mechanisms regulating NRF2 stability have also been reported. Here we employed an HTS approach and identified a small molecule, BC-1901S, that stabilized NRF2 and increased its activity. BC-1901S activated NRF2 by inhibiting NRF2 ubiquitination in a KEAP1-independent manner. It further increased NRF2-dependent anti-oxidant gene transcription, and exhibited anti-inflammatory effects *in vitro* and *in vivo*. Further, we identified a new NRF2-interacting partner, DDB1 and CUL4 Associated Factor 1 (DCAF1), an E3 ligase that targeted NRF2 for proteasomal degradation. Mechanistically, BC-1901S directly bound to DCAF1 and disrupted NRF2/DCAF1 interaction, thus activating NRF2. These findings provide new insights in NRF2 biology and NRF2 based anti-inflammatory therapy.

## Highlights

• A small molecule BC-1901S was identified as NRF2 activator based on high-throughput screening.
• BC-1901S activates NRF2 through KEAP1-independent pathway and exhibit anti-inflammatory and anti-oxidant properties*.*
• BC-1901S potentially disrupts the interaction between NRF2 and its novel E3 ligase subunit DCAF1.
• An unbiased high-throughput approach that directly measures the level of NRF2 protein in cells was used.

## Graphical abstract

**Figure:**
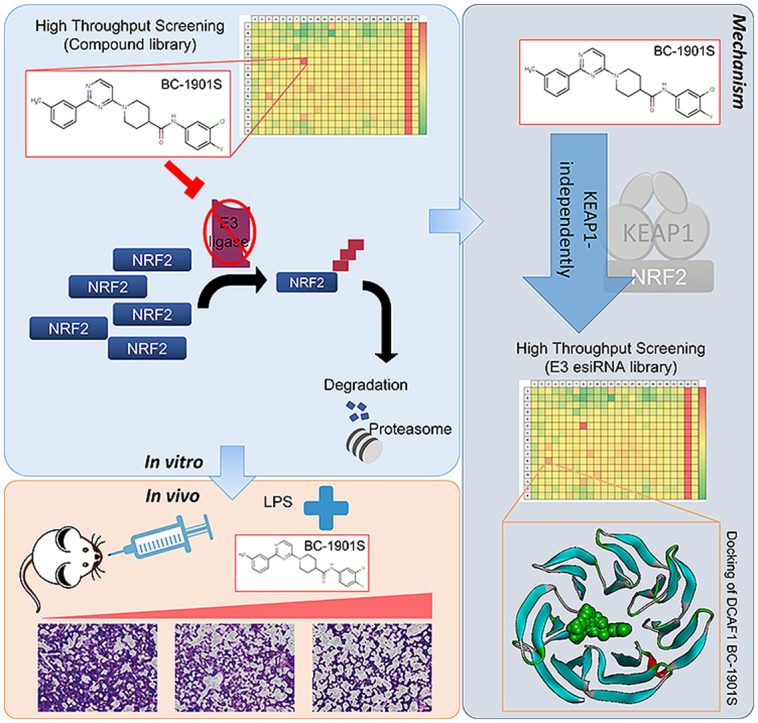

## 1 Introduction

Nuclear factor erythroid 2-related factor 2 (NRF2) is a master transcription factor controlling cellular anti-oxidant responses [1,2]. NRF2 also regulates the magnitude of innate immune responses in several disease models, including pneumonia; NRF2 deletion exacerbated inflammatory lung injury, while NRF2 activation was protective [[3], [4], [5], [6], [7], [8], [9]]. Mechanistically, NRF2 activation is repressed in homeostatic conditions by association with the protein subunit Kelch-like ECH-Associated Protein 1 (KEAP1) [10]. KEAP1 is an adapter protein in an E3 ubiquitin ligase complex involving CUL3 and RBX1, and directs the ubiquitination and degradation of NRF2 in the proteasome. In the presence of electrophiles generated by oxidative stress, KEAP1 dissociates with NRF2, increasing NRF2 protein abundance. NRF2 translocates to the nucleus and facilitates transcriptional up-regulation of several anti-oxidant response element (ARE) genes [1,11]. In addition to KEAP1-dependent regulation, NRF2 is also subjected to regulation through the ubiquitin-proteasome system by KEAP1-independent mechanisms. The E3 ligases DCAF11 [12], HRD1 [13], and β-TrCP [14] also interact with NRF2 and regulate its abundance through ubiquitination and degradation in the proteasome.

The ubiquitin proteasome system (UPS) is a cellular process that controls protein degradation. Ubiquitin (Ub), a small 76 amino acid protein, is conjugated to substrate proteins in a process that involves Ub-activating enzymes (E1), Ub-conjugating enzymes (E2), and Ub ligases (E3) [15,16]. Ubiquitin conjugation serves several roles, but primarily directs proteins to the proteasome for degradation. Several E3 ligases have been shown to regulate inflammatory pathways in lung, usually indirectly by controlling abundance of their corresponding substrates [[17], [18], [19], [20], [21], [22]].

NRF2 critically regulates the magnitude of innate immune responses in acute lung injury. In humans, functional NRF2 polymorphisms increase the risk of acute lung injury and acute respiratory distress syndrome (ARDS) [23]. In murine models of acute lung injury, NRF2 deletion results in greater pulmonary inflammation in response to non-lethal doses of intra-tracheal LPS, including increased NF-κB activity and secretion of TNFα [7]. Global lung transcriptomic analysis has revealed NRF2 KO mice have increased pro-inflammatory signaling following intra-peritoneal LPS treatment as compared to WT mice, including the cytokines TNFα and IL-6, and chemokines MIPs, MIG, KC, and ITAc. Conversely, NRF2 activation is protective in models of acute lung injury, including hyperoxia [3,8] and *S. aureus* pneumonia [5]. Thus, NRF2 is a critical and central factor in determining the magnitude of innate immune responses during acute lung injury. Small molecule activators of NRF2 are a promising approach for mitigating excessive innate immune responses.

NRF2 protein stability is a key mechanism regulating NRF2 activation. Many prior screens for NRF2 activators have generally focused on disrupting the KEAP1/NRF2 protein-protein interaction (PPI). Here, we deployed an unbiased approach that directly measures the level of NRF2 protein in cells using Nanoluciferase technology. Using a compound library focused on PPI disruption, we identified the compound BC-1901S as a potent NRF2 activator. We show BC-1901S activated NRF2, and exhibited strong anti-inflammatory and anti-oxidant properties *in vitro*. BC-1901S was also protective in a murine model of LPS-induced lung injury by reducing the magnitude of inflammatory responses. Further, we observed BC-1901S activated NRF2 through KEAP1-independent pathway, and through RNAi screening we uncovered the ubiquitin E3 ligase DDB1 and CUL4 Associated Factor 1 (DCAF1) as a non-canonical regulator of NRF2 stability. Lastly, we show the BC-1901S mechanism of action is potentially mediated through disrupting the DCAF1/NRF2 axis, describing a new layer of NRF2 control.

## 2 Results

**Fig. 1:**
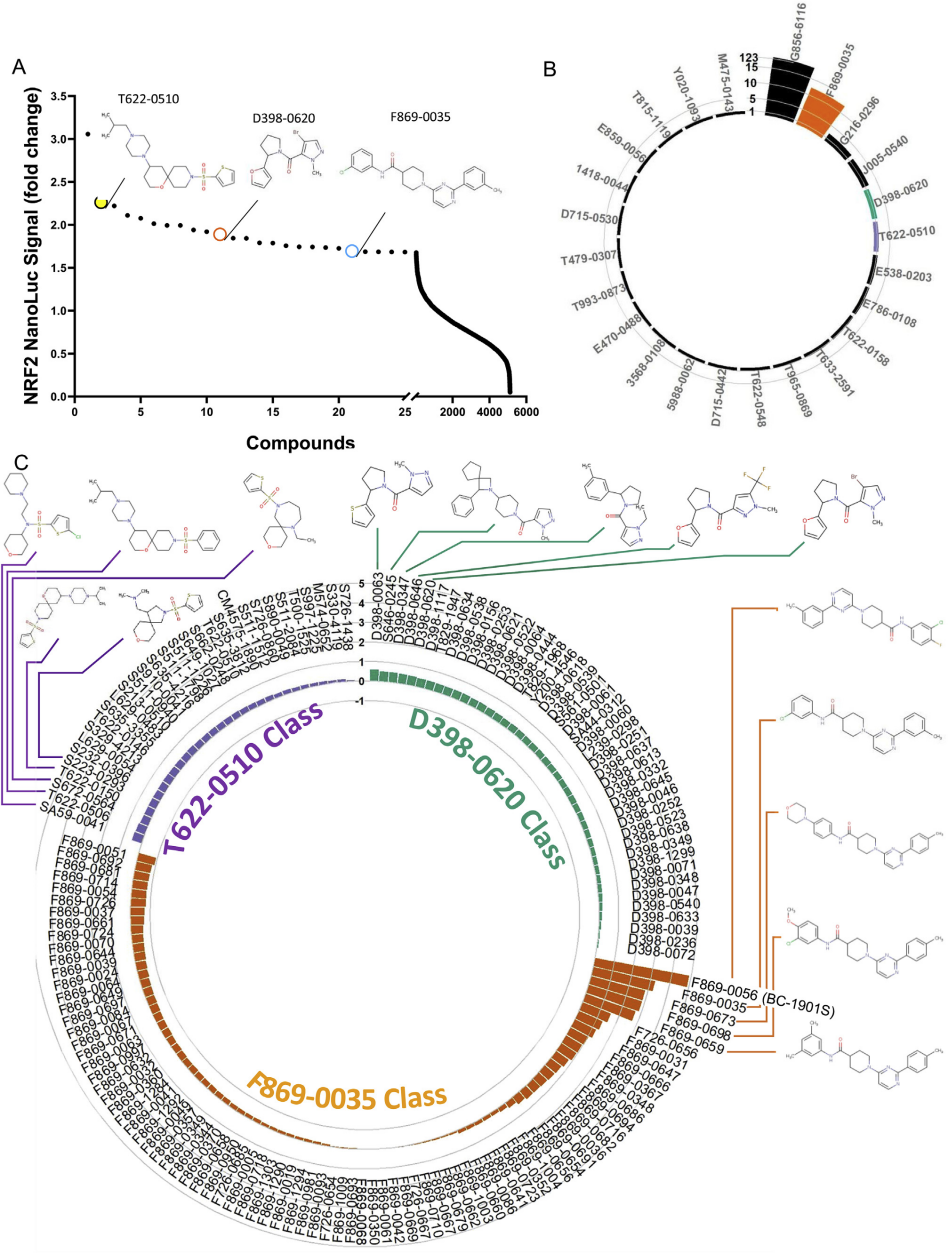
High throughput screen of NRF2 stabilizers. A) Beas-2B cells stably expressing NRF2 Nano-Luc were screened with a small molecule library (ChemDiv, ~5000 cpds, 10 μM). Compounds (100 nl) were dispensed into Thermo 384 well plate, before cells (5000 per well) were added. 18h later, Nano-luciferase reagent were added and luminescence signals were collected and quantified using CLARIOstar plate reader. B) Confirmatory screening of the top 0.5% of compounds at 25 μM. Circular barplot represents log(2)-transformed NRF2 Nano-Luc signal of the top 25 compounds from primary screen. C) Circular barplot represents log(2)-transformed NRF2 Nano-Luc signal of the Analog/derivative(s) of F869-0035, D398–0620 and T622-0510 compounds tested at 25μΜ in Beas-2B cells stably expressing NRF2 Nano-Luc.

*A High-throughput screen identifies several novel NRF2 activators.* There have been several strategies to screen for NRF2-activating compounds [[24], [25], [26]]. To uncover potential NRF2 activators in lung cells, we deployed an unbiased approach directly measuring NRF2 stability, which is critically related to NRF2 function and downstream effects. We created a stable cell line expressing a CMV promoter-driven NRF2 Nano-Luc fusion protein (Promega) in human bronchial epithelial cells (Beas-2B), which allowed quantitative measurement of NRF2 protein abundance. We first tested the assay robustness by measuring the Z′-factor. Cells had low baseline NRF2 Nano-Luc expression, but after treatment with the proteasome inhibitor MG132, NRF2 Nano-Luc intensity increased. The Z′-factor for control vs. MG132-treated cells was 0.51, which was adequate for carrying out HTS(Fig. S1). We then screened a chemical library (ChemDIV) consisting of ~5000 compounds designed to disrupt protein-protein interactions for the ability to increase stably-expressed NRF2 Nano-Luc abundance (Fig. 1A and Table 1). We selected the top 0.5% (n = 24) of compounds from our initial screen and performed secondary validation assays at several different doses (Table S1). At a dose of 25 μM, we identified four compounds that increased NRF2 Nano-Luc intensity most robustly – G856–6116, F869-0035, D398–0620 and T622-0510 (Fig. 1B and Table S1). Notably, any compound interfering with the UPS, including downstream proteasome inhibitors should increase NRF2 abundance. Indeed, a top hit in the initial screen, G856-6116 (Fig. S2A), increased NRF2 Nano-Luc signal most robustly, but was identified as a proteasome inhibitor. While G856-6116 increased both NRF2 Nano-Luc signal and endogenous NRF2 abundance, it also increased total cellular protein poly-ubiquitination, as measured by ubiquitin-K48-linkage-specific immunoblotting, the canonical signal for proteasomal degradation (Figs. S2A–B). Further, G856-6116 directly inhibited proteasome activity *in vitro*, leading us to conclude its effect on NRF2 was non-specific (Fig. S2C).

**Table 1:** The list of top 0.5% of compounds from the primary screen in Fig. 1A.

Compound	Fold	Compound	Fold
G856-6116	3.058	T479-0307	1.843
T622-0510	2.263	T633-2591	1.790
T622-0158	2.220	E470-0488	1.787
3568-0108	2.110	1418-0044	1.757
E859-0056	2.076	J005-0540	1.742
Y020-1093	2.013	E538-0203	1.735
T622-0548	1.993	D715-0442	1.726
G216-0296	1.993	F869-0035	1.694
T815-1119	1.940	M475-0143	1.686
T965-0869	1.920	T993-0873	1.685
D398-0620	1.890	D715-0530	1.683
E786-0108	1.844	5988–0062	1.682

**Fig. 2:**
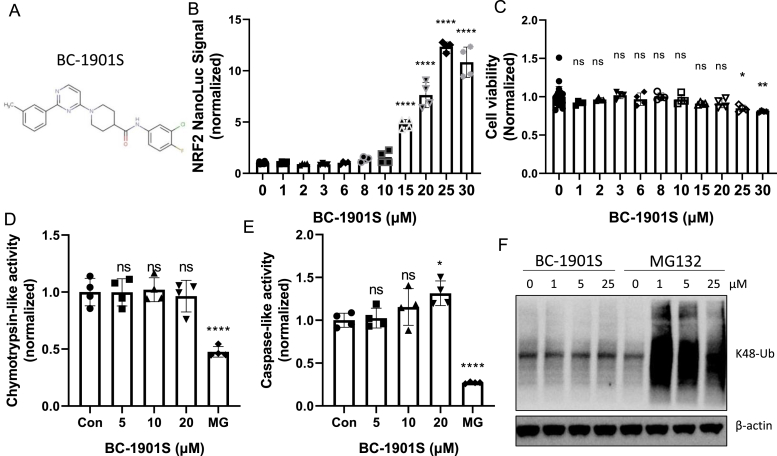
BC-1901S is a proteasome-independent NRF2 stabilizer. A) Structure of BC-1901S. B) BC-1901S dose course study in NRF2 Nano-Luc cells after 16 h treatment. Data and mean ± SD of 3 independent experiments. C) Cell viability assay (MTT) with BC-1901S dose course after 16 h. Data and mean ± SD of 3 independent experiments. D, E) Chymotrypsin-like (D) and Caspase-like activity (E) (normalized) after 16 h BC-1901S treatment in Beas-2B cells. Data and mean ± SD of 3 independent experiments. F) Immunoblot analysis of total K-48 ubiquitinated proteins after 16 h BC-1901S or MG132 treatment. ns = not significant; P < 0.05, *; P < 0.01, **; P < 0.001, ***; P < 0.0001, ****; by one-way ANOVA with adjusted P-value for multiple comparisons (Dunnett's) compared to control or “0”.

*BC-1901S activates NRF2 upstream of the proteasome:* We further analyzed the top hits through their chemical scalability and screened analogs of compounds T622-0510, D398–0620 and F869-0035 for effect on NRF2 Nano-Luc activation (Fig. 1C). We identified the compound F869-0056 (BC–1901S) as a strong NRF2 Nano-Luc activator, and chose it as a lead compound (Fig. 2A). We observed a dose-dependent increase in NRF2 Nano-Luc signal with BC-1901S treatment (Fig. 2B), without affecting cellular viability (Fig. 2C). To further define the mechanism of BC-1901S mediated NRF2 activation, we first asked whether this compound interfered with proteasome activity. BC-1901S did not affect Chymotrypsin-like activity (Fig. 2D) or Caspase-like activity assays (Fig. 2E) compared to the proteasome inhibitor MG132. Further, while MG132 increased total cellular K48-linked poly-ubiquitinated proteins, BC-1901S had no effect on K48-poly-ubuiqitination (Fig. 2F), suggesting that BC-1901S activates NRF2 independent of the proteasome. These results suggest BC-1901S potentially activates NRF2 by inhibiting its ubiquitination and subsequent degradation.

**Fig. 3:**
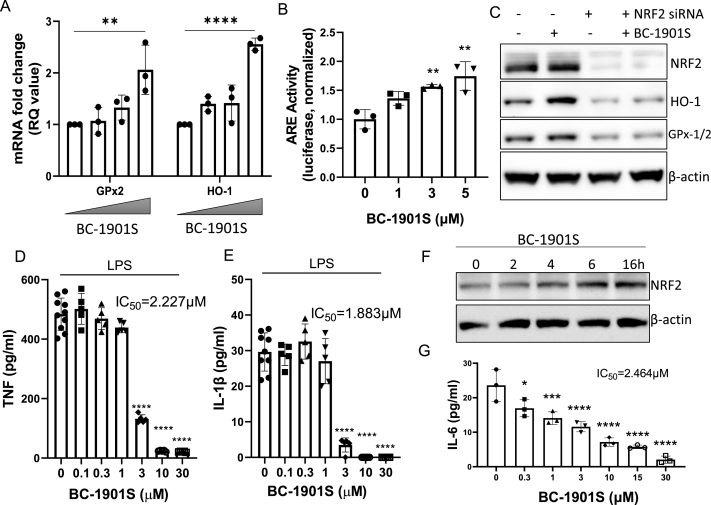
BC-1901S increases NRF2-dependent gene transcription and decreases inflammation. A) RT-qPCR of GPx2 and HO-1 in Beas-2B cells treated with BC-1901S at 0, 0.1, 1, and 10 μM. Fold change (RQ) is normalized to endogenous GAPDH expression. Data and mean ± SD of 3 independent experiments. B) Beas-2B cells were transfected with an ARE-reporter (pGL4.37[luc2P/ARE/Hygro]) vector and treated with BC-1901S at various doses. Luminescence signals were collected and quantified. Data and mean ± SD of 3 independent experiments. C) NRF2, HO-1, GPx-1/2, and β-Actin immunoblots after NRF2 dsiRNA knockdown with or without BC-1901S treatment. D, E) PBMCs were treated with LPS or LPS and BC-1901S at various doses for 18h. Cell culture supernatants were then assayed for TNF (D) and IL-1β (E) using ELISA. Data and mean ± SD of 3 independent experiments. F) Immunoblot of NRF2 in MLE-12 after treatment with BC-1901S at indicated time points. G) MLE were treated with LPS or LPS and BC-1901S at various doses for 18h. Cell culture supernatants were then assayed for IL-6 using ELISA. Data and mean ± SD of 3 independent experiments. P < 0.05, *; P < 0.01, **; P < 0.001, ***; P < 0.0001, ****; by one-way ANOVA with adjusted P-value for multiple comparisons (Dunnett's) compared to “0”.

*BC-1901S increases NRF2-dependent gene transcription and reduces inflammatory cytokine release in vitro.* Since we identified BC-1901S as a potential NRF2 activator, we tested downstream effects in Beas-2B cells. Treatment with BC-1901S increased the gene expression of the NRF2-transcription targets *Gpx2* and *HO-1* in a dose-dependent manner (Fig. 3A) as well as increasing ARE reporter activity (Fig. 3B). BC-1901S also increased protein levels of NRF2, HO-1, and GPx-1/2; this effect was diminished upon NRF2 knockdown, suggesting BC-1901S activity proceeds through NRF2 (Fig. 3C). In addition to directly regulating ARE genes, NRF2 also has anti-inflammatory effects and has been shown to reduce proinflammatory cytokine release from a number of different cell types [6,27,28]. BC-1901S exhibited broad anti-inflammatory properties in LPS-stimulated human PBMCs, dose dependently inhibiting release of several proinflammatory cytokines and chemokines, including TNF (Fig. 3D), IL-1β (Fig. 3E), IL-8, MIP-1β and IL-1α (Fig. S3). We further tested BC-1901S in murine lung epithelial cells (MLE12), which respond to pro-inflammatory stimuli and secrete several innate immune cytokines, including Interleukin-6 (IL6) [18]. BC-1901S increased NRF2 abundance in MLE12 cells in a time dependent manner (Fig. 3F), and also inhibited release of IL-6 in LPS-stimulated cells (Fig. 3G).

**Fig. 4:**
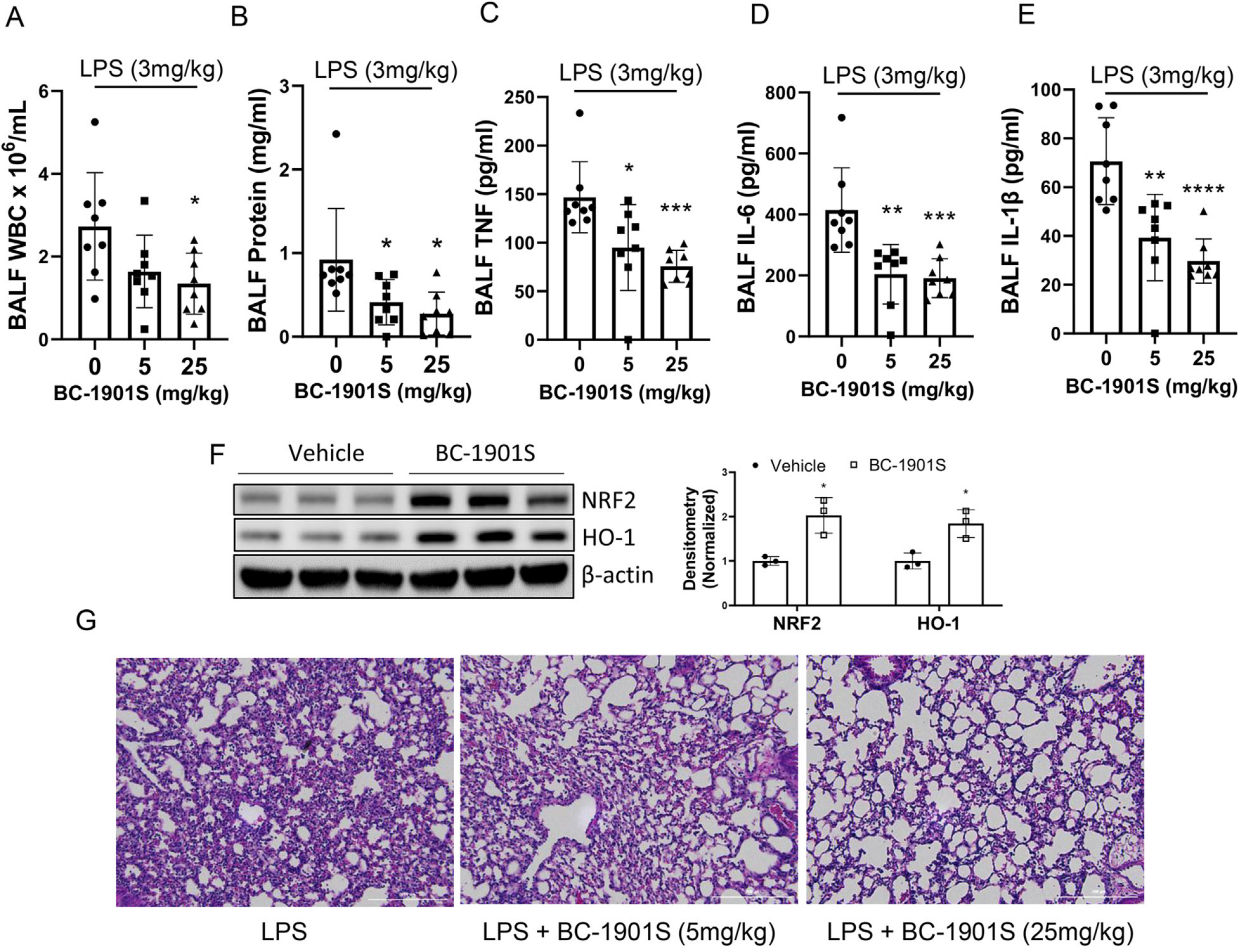
BC-1901S ameliorates LPS-induced pulmonary inflammation. C57BL/6J mice (n = 8 per group) were treated with intra-tracheal LPS (3 mg/kg) and either control (corn oil, i.p.) or BC-1901S at 5 mg/kg or 25 mg/kg (i.p.). 18h later, mice were euthanized and A) Broncho-alveolar lavage fluid (BALF) WBC count, B) BALF protein concentrations, C-E) BALF TNF, IL-6 and IL-1β were measured. Data represent mean ± SD. (F). Representative immunoblots and densitometry for NRF2, HO-1, and β-Actin from lung homogenates from LPS or LPS/BC-1901S (25 mg/kg) co-treated mice. Data represent mean ± SD. G) Representative lung histological images (H&E staining) from LPS and LPS/BC-1901S co-treated mice. P < 0.05, *; P < 0.01, **; P < 0.001, ***; P < 0.0001, ****; by one-way ANOVA with adjusted P-value for multiple comparisons (Dunnett's) compared to control or “0.”

*BC-1901S is anti-inflammatory in a murine model of LPS-induced acute lung injury.* Activation of the NRF2 pathway is protective in several models of murine acute lung injury [4,6,8,29]. We sought out to determine if BC-1901S might similarly be protective in a model of LPS-induced murine lung injury. We used a non-lethal dose of intratracheal LPS to test whether BC-1901S would reduce inflammatory responses in the lung and reduce the severity of lung injury. Mice were divided into three groups (n = 8 mice per group): LPS treatment (3 mg/kg intra-tracheal), LPS + BC-1901S 5 mg/kg (intra-peritoneal), and LPS + BC-1901S 25 mg/kg (intra-peritoneal). BC-1901S reduced broncho-alveolar lavage fluid (BALF) WBC counts, protein concentrations, and pro-inflammatory cytokine levels of TNF, IL-6 and IL-1β in a dose dependent manner relative to vehicle (Fig. 4A–E). Immunoblots from lung homogenates showed BC-1901S at 25 mg/kg significantly increased NRF2 and HO-1 levels compared to LPS-treated mice (Fig. 4F). Lastly, BC-1901S also reduced the immune cells infiltration by tissue histology (Fig. 4G). Taken together, these results suggest that BC-1901S is protective in LPS-induced lung injury, potentially through activation of NRF2 protein and activity.

**Fig. 5:**
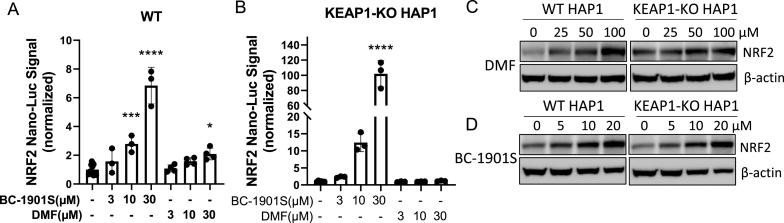
BC-1901S increases NRF2 abundance in a KEAP1-independent manner. A, B) NRF2 Nano-Luc stably expressed HAP1 WT (A) or KEAP1 KO (B) cells were treated with various concentrations of DMF or BC-1901S for 16 h. Nano-luciferase reagent were then added and luminescence signals were collected and quantified. Data and mean ± SD of 3 independent experiments. C, D) Immunoblots for NRF2 and β-Actin in WT and KEAP1-KO cells treated with DMF or BC-1901S in a dose course. P < 0.05, *; P < 0.001, ***; P < 0.0001, ****; by one-way ANOVA with adjusted P-value for multiple comparisons (Dunnett's) compared to control.

*BC-1901S activates NRF2 independent of KEAP1.* We determined BC-1901S stabilized NRF2 abundance upstream of the proteasome and thus its effect may be through modulation of E3 ligase activity. The E3 ligase KEAP1 is the canonical E3 ligase controlling NRF2 abundance [10]. Additionally, E3 ligases DCAF11, HRD1, and β-TrCP also regulate NRF2 independent of KEAP1 [[12], [13], [14],^30^]. Thus, there are several overlapping, partially redundant mechanisms controlling NRF2 protein level through ubiquitination and proteasomal degradation. We tested whether BC-1901S increased NRF2 protein level in KEAP1-knockout cells. Using KEAP1 WT and KO HAP1 cells as background, we generated cell lines stably expressing NRF2 Nano-Luc. We observed the KEAP1 inhibitor DMF only increased NRF2 Nano-Luc signal in WT cells, however BC-1901S dose-dependently increased NRF2 Nano-Luc signal in both WT and KEAP1-KO cells, suggesting KEAP1 is not essential for BC-1901S effect (Fig. 5A, B). We also examined endogenous NRF2 by immunoblot in both WT and KEAP1-KO cells after BC-1901S treatment and observed that NRF2 similarly accumulated with drug treatment in both WT and KEAP1-KO cells. However, KEAP1 inhibitor dimethyl fumarate (DMF) only induced NRF2 activation in WT but not KEAP1-KO cells (Fig. 5C–D). Thus, BC-1901S increases NRF2 abundance through pathway independent of KEAP1.

**Fig. 6:**
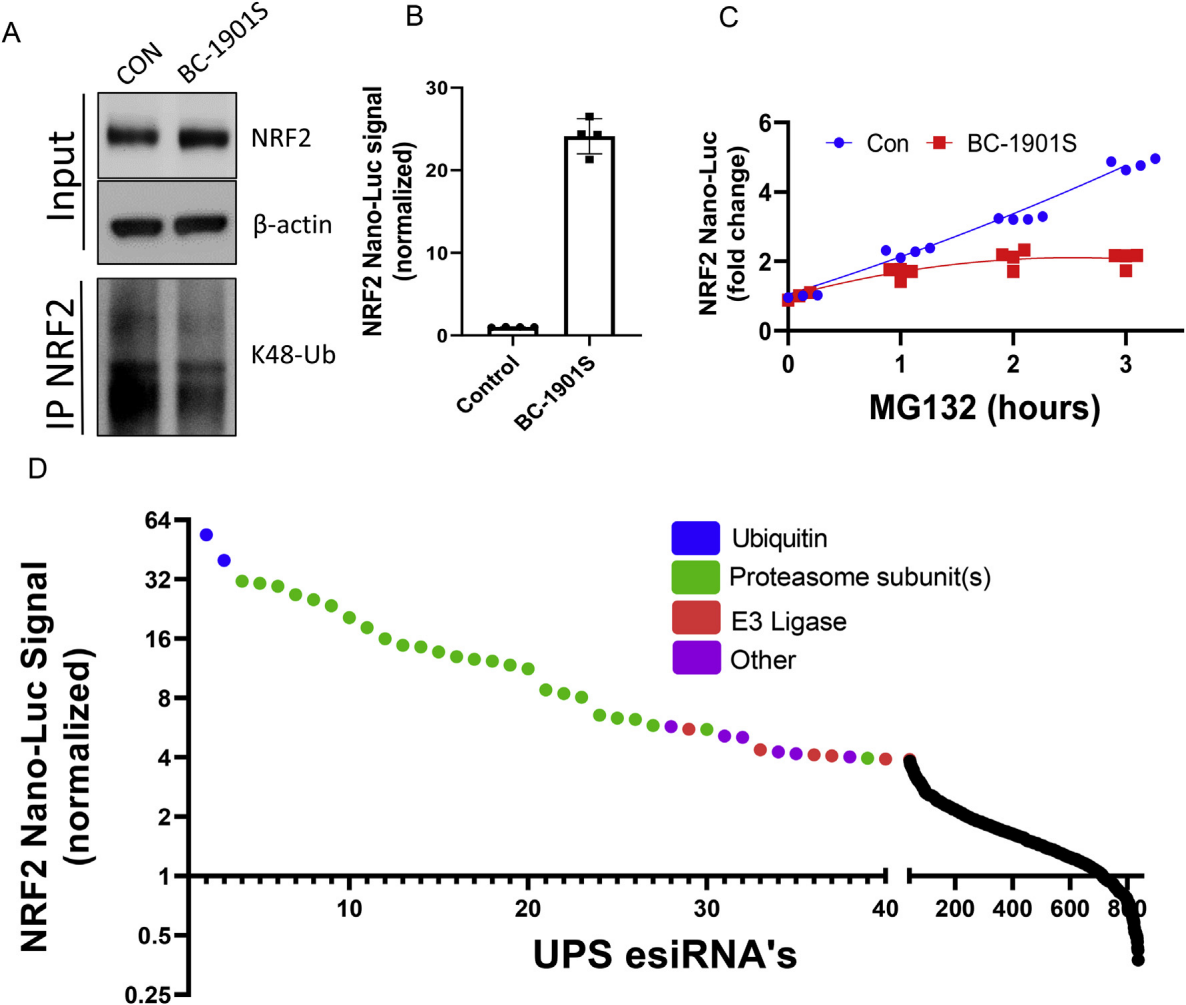
BC-1901S decreases NRF2 ubiquitination. A) Beas-2B cells were treated BC-1901S for 16 h followed by NRF2 immunoprecipitation and immunoblot for K48-Ub. B) Normalized NRF2 Nano-Luc intensity after 16 h BC-1901S treatment, prior to addition of MG132. Data and mean ± SD of 4 independent experiments. C) Saturation curves of NRF2 Nano-Luc intensity after addition of MG132 to control of BC-1901S pre-treated cells. Data and mean ± SD of 4 independent experiments. D) NRF2 Nano-Luc intensity (normalized) 72 h after treatment with a UPS esiRNA library (~800 proteins). The horizontal axis of top 40 hits is broadened for better visualization. Blue dots = Ubiquitin esiRNA's, green dots = Proteasome subunit esiRNA's, red dots = E3 Ligase esiRNA's, purple dots = other esiRNA's. (For interpretation of the references to colour in this figure legend, the reader is referred to the Web version of this article.)

*A ubiquitin-proteasome system esiRNA screen reveals several potential NRF2-regulating factors*. Since BC-1901S increased the abundance of NRF2 upstream of the proteasome, and the major mechanism regulating NRF2 abundance is NRF2 ubiquitination, we sought out to determine if BC-1901S affects NRF2 ubiquitination. Beas-2B cells were treated with BC-1901S and NRF2 ubiquitination was measured by Co-immunoprecipitation (IP) with anti-NRF2 followed by K48-Ubiquitin immunoblotting. BC-1901S treatment increased NRF2 protein in total cell lysate but reduced poly-ubiquitinated NRF2, suggesting an effect on NRF2 ubiquitination (Fig. 6A). As a complementary approach, we asked whether pre-treatment with BC-1901S would affect the degree of NRF2 activation upon proteasomal inhibition, due to the effect of BC-1901S decreasing the overall pool of ubiquitinated NRF2. We treated NRF2 Nano-Luc Beas-2B cells overnight with BC-1901S and assayed NRF2 Nano-Luc intensity. Cells treated with BC-1901S had a >20-fold increase in NRF2 Nano-Luc intensity compared to control (Fig. 6B). Then, following overnight control or BC-1901S treatment, we treated with MG132 and assayed NRF2 Nano-Luc intensity at 1, 2, and 3 h, normalizing the signal fold increase to 0hr. While the NRF2 Nano-Luc signal increased robustly with MG132 time course in control pre-treated cells, we observed a decreased rate of NRF2 Nano-Luc accumulation in BC-1901S pre-treated cells (Fig. 6C). This experiment suggests that BC-1901S blocks the ubiquitin conjugation step of the NRF2 degradation pathway. Since NRF2 ubiquitination is essential for its turnover at the proteasome, blocking NRF2 ubiquitination would prevent its accumulation upon proteasome inhibition by decreasing the pool of ubiquitinated NRF2 available. Thus, we hypothesized that BC-1901S might modify E3 ligase interactions with NRF2 independent of KEAP1.

Using Beas-2B cells stably expressing NRF2 Nano-Luc, we then performed a high-throughput screen of esiRNAs targeting ~800 components of the ubiquitination proteins (Ubiquitin, proteasome subunits, E1, E2, E3, De-ubiquitinases (DUBs), etc.) to determine the relevant UPS components regulating NRF2 abundance. Knockdown of ubiquitin (blue dots) or proteasome subunits (green dots) increased NRF2 Nano-Luc signal most robustly, but we also observed that knockdown of several E3 ligases increased NRF2 abundance robustly (red dots) (Fig. 6D and Table S3).

**Fig. 7:**
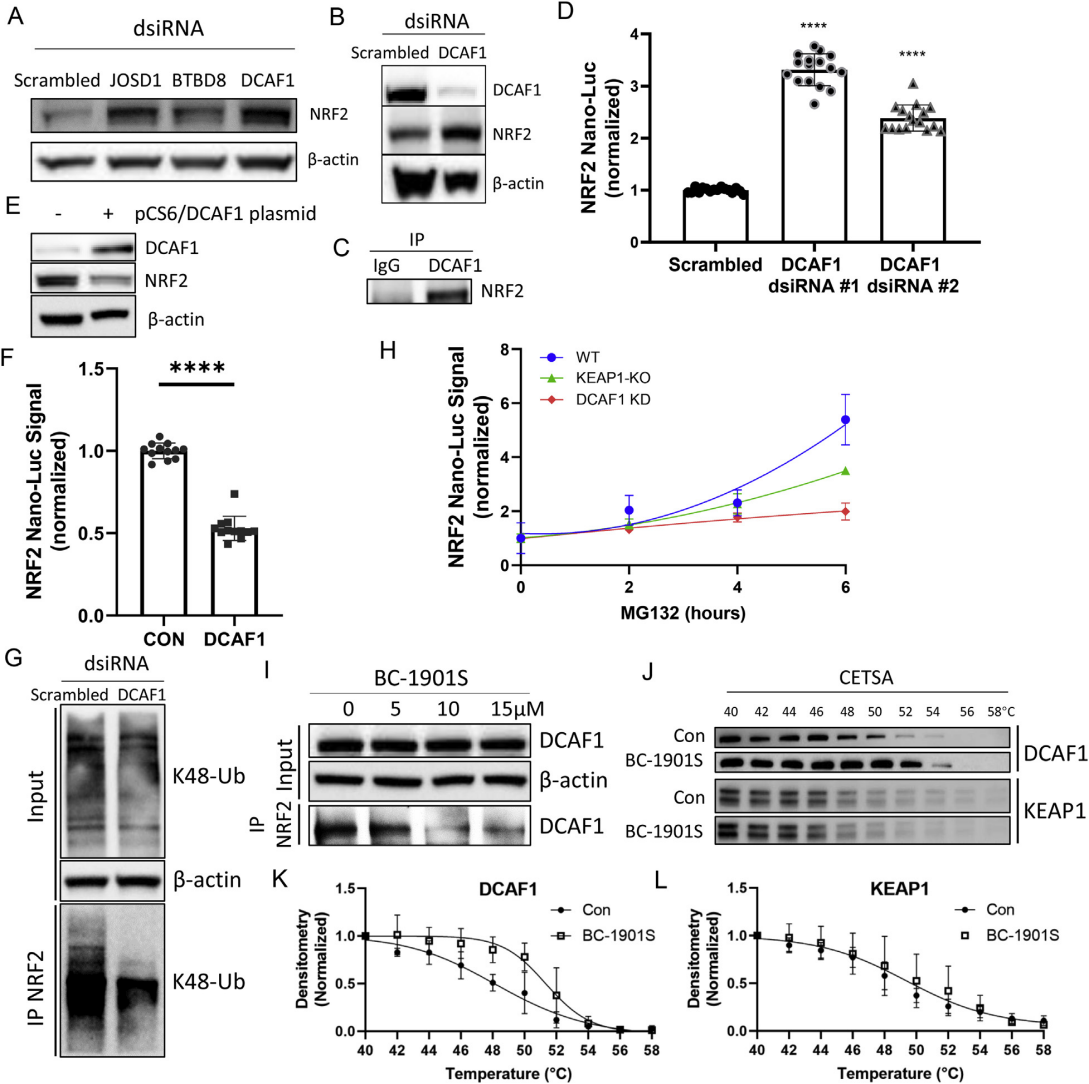
DCAF1 directs NRF2 for ubiquitination and proteasomal degradation and is the potential target of BC-1901S. A) Immunoblots for NRF2 and β-Actin in Beas-2B cells after knockdown of top-hit E3 ligases JOSD1, BTBD8, or DCAF1 using dsiRNA. B) Immunoblots for DCAF1, NRF2, and β-Actin after DCAF1 knockdown. C) Association between DCAF1 and NRF2 by Co-IP. D) NRF2 Nano-Luc intensity (normalized) after DCAF1 knockdown using two independent dsiRNAs. Data and mean ± SD of 3 independent experiments. E) Immunoblots of DCAF1 and NRF2 expression in after DCAF1 overexpression. F) NRF2 Nano-Luc intensity (normalized) after DCAF1 overexpression in Beas-2B cells. Data and mean ± SD of 3 independent experiments. G) Immunoblots of Beas-2B cells treated with scrambled or DCAF1 dsiRNA followed by IP for NRF2 and IB for K48-Ubiquitin. H) NRF2 Nano-Luc intensity (normalized) in WT, DCAF1-KD, or KEAP1 KO HAP1 NRF2 Nano-Luc cells followed by treatment with MG132 for various time-points. Data and mean ± SD of 3 independent experiments. I) NRF2 binding assay where Beas-2B cells were treated with increasing concentrations of BC-1901S, followed by IP for NRF2 and IB for DCAF1. J) Cell based thermal shift assay (CETSA) of DCAF1 and KEAP1 proteins in Beas-2B cells treated with BC-1901S or control. Blots are representative of 3 independent experiments. K, L) Quantification of DCAF1 and KEAP1 abundance from CETSA experiments. P < 0.05, *; P < 0.0001, ****; by one-way ANOVA with adjusted P-value for multiple comparisons (Dunnett's) compared to control, except indicated specifically.

*E3 ligase adapter protein DCAF1 regulates NRF2 abundance*. We confirmed that silencing of several of the “top-hit” proteins (JOSD1, BTBD8, and DCAF1) from the screen also increased endogenous NRF2 by immunoblotting in Beas-2B cells, (Fig. 7A, B). We noted that one protein – DCAF1 – belonged to a family of adapter proteins for the CUL4A-RBX1-DDB1 E3 ligase complex, and that another member of this family (DCAF11) has been identified in regulating NRF2 stability [12]. Thus, we sought out to determine if DCAF1 might also regulate NRF2 stability. We first confirmed NRF2/DCAF1 interaction by co-immunoprecipitation (Fig. 7C). DCAF1 knock-down using two independent dsiRNA's also increased NRF2 Nano-Luc signal (Fig. 7D); conversely, DCAF1 over-expression reduced endogenous NRF2 (Fig. 7E) and NRF2 Nano-Luc signal (Fig. 7F). We then tested whether DCAF1 knockdown would reduce poly-ubiquitinated NRF2. Cells were treated with *Dcaf1* dsiRNA and NRF2 ubiquitination was measured by Co-IP with anti-NRF2 followed by K48-Ubiquitin immunoblot. Less K48-Ub NRF2 was detected after *Dcaf1* knockdown vs. scrambled control (Fig. 7G). As DCAF1 affected NRF2 ubiquitination, we further hypothesized that DCAF1 knockdown would reduce flux of ubiquitinated NRF2 to the proteasome and thus render cells resistant to NRF2 accumulation with MG132 treatment. In WT HAP1 cells stably expressing NRF2 Nano-Luc, we observed a robust time-dependent increase in NRF2 Nano-Luc intensity over 6 h of MG132 treatment, an effect blunted by DCAF1 knockdown; further, the effect of DCAF1 knockdown in inhibiting NRF2 accumulation was more robust than the effect observed in KEAP1 KO cells (Fig. 7H). This suggests that DCAF1 is a more relevant E3 ligase regulating NRF2 stability compared to KEAP1 in HAP1 cells.

Lastly, we tested whether BC-1901S could disrupt the interaction between NRF2 and DCAF1. We first took an *in-silico* modeling approach generated a homology model of DCAF1 and determined that BC-1901S can potentially directly interact with DCAF1 (Fig. S4). BC-1901S fit nicely within the DCAF1 pocket and exhibited multiple interactions with DCAF1 residues. Further, in cell-based binding assays, we observed that increasing doses of BC-1901S disrupted interaction between DCAF1 and NRF2, as less DCAF1 was detected after NRF2 IP. (Fig. 7I). Finally, as an orthogonal approach, we performed cellular thermal shift assays (CETSA) [31] using BC-1901S. Cells were treated with control (DMSO) or BC-1901S and lysates were equally fractioned into 10 aliquots and subjected to a range of increasing temperatures to promote protein denaturation. We hypothesized that DCAF1 would demonstrate resistance to heat-induced protein denaturation should it interact with BC-1901S, with BC-1901S raising the theoretical thermal stability curve of DCAF1. After high-speed spin to precipitate heat-denatured proteins, the soluble protein fraction from each temperature was then immunoblotted for DCAF1 or KEAP1. BC-1901S increased the temperature at which DCAF1 transitioned to an insoluble aggregate, relative to control treatment (Fig. 7J–K). As a control, we blotted for KEAP1, which showed no change in its thermal denaturation profile upon BC-1901S treatment (Fig. 7J, L). Thus, BC-1901S may directly interact with DCAF1, but not KEAP1 (Fig. 7K-L).

## 3 Discussion

NRF2 is a central regulator of cellular anti-oxidant and inflammatory responses, with a vast volume of literature describing its regulation and potential applicability to treat human disease [1,2,7,28]. We uncovered a novel mechanism of NRF2 protein regulation and developed a new small molecular activator of NRF2 protein to affect inflammatory signaling. In this study, our main findings are: 1) BC-1901S is a novel NRF2 activator that prevents NRF2 protein degradation, 2.) BC-1901S exhibits anti-inflammatory effects in a murine model of LPS-induced acute lung injury, 3) BC-1901S operates in a KEAP1-independent manner, 4) Identification of DCAF1 as a new and potentially dominant NRF2 repressor, and 5) BC-1901S may function through inhibition of the DCAF1-NRF2 degradation axis. These findings have potential for many applications where BC-1901S could be used as a NRF2 activator and anti-inflammatory agent.

Drug discovery efforts to activate NRF2 protein have focused on interference of the KEAP1/NRF2 interaction. Electrophiles are known KEAP1/NRF2 disruptors, leading to NRF2 activation, but these species suffer from off-target effects [24,25]. Additionally, several non-electrophilic compounds have been described that inhibit KEAP1/NRF2 interactions, through non-covalent or domain-specific targeting [2,24,32,33]. Here we utilized quantitative luminescence to directly measure NRF2 protein abundance rather than NRF2-dependent responses such as ARE activity or transcriptional changes of NRF2 targets. This system facilitated unbiased screening for novel direct regulators of NRF2 protein level. The platform has broad applicability that could be utilized to screen for other factors influencing NRF2 abundance (miRNA's, lncRNA's, metabolites, small molecules, etc.)

KEAP1 is recognized as the canonical E3 ligase regulating NRF2 protein ubiquitination and degradation. However, in addition to canonical KEAP1-mediated regulation, NRF2 is also subject to regulation in the UPS via several other E3 ligases, including DCAF11, HRD1, and β-TrCP. As our lead NRF2 compound BC-1901S showed activity even in KEAP1 KO cells, we investigated if another ubiquitin E3 ligase regulated NRF2 stability. In the course of BC-1901S development, we uncovered a novel mechanism of NRF2 proteolytic regulation by the ubiquitin E3 ligase DCAF1. DCAF1 potently controls NRF2 stability in Beas-2B and HAP1 cells, and DCAF1 knockdown results in decreased NRF2 ubiquitination. Further, DCAF1 may even be a more potent regulator of NRF2 protein stability than KEAP1, as we observed DCAF1 knockdown impaired NRF2 Nano-Luc accumulation upon proteasome inhibition to significantly greater extent than KEAP1 knockout (Fig. 7H). If KEAP1 was the primary mediator of NRF2 ubiquitination, its knockout should preclude all ubiquitination of NRF2, yet we observe DCAF1 depletion significantly affected NRF2 stability and accumulation. Thus, given the centrality of NRF2 in controlling cellular antioxidant and anti-inflammatory responses, there appear to be several distinct layers of mechanism controlling NRF2 abundance. Further research is needed to determine the master regulator of NRF2 stability.

Our studies identify BC-1901S, which increased NRF2 abundance and NRF2-dependent anti-inflammatory signaling. Strategies to boost NRF2 activity may be beneficial in settings of acute oxidative and inflammatory stress, but the negative consequences of chronic NRF2 activation are also well described. For example, enhanced NRF2 expression is associated with chemoresistance in several malignancies, and NRF2 activity can promote a growth advantage to malignant cells [34,35]. Our strategy using BC-1901S indirectly increases NRF2 activity by reducing its shuttling to the proteasome and would likely have only a transient effect on NRF2 activity. However, further toxicological and pharmacological studies of BC-1901S are warranted to determine its properties *in vivo*. In addition, our platform could easily be adapted to identify small molecules or proteins that negatively influence NRF2 abundance, which could be tested in cells or disease states (malignancies) where chronic NRF2 over-expression is deleterious.

In summary, our data provide evidence that the compound BC-1901S is a NRF2 activator and has anti-inflammatory properties *in vitro* and *in vivo* in a model of LPS-induced acute lung injury. We show a new mechanism of NRF2 control through the E3 ligase DCAF1, and propose a model in which BC-1901S directly binds to E3 ligase subunit DCAF1, disrupts the interaction between NRF2 and DCAF1, and reduces NRF2 ubiquitination and degradation to increase its activity. These findings have implications for therapy in disease states where transient NRF2 activation could blunt the deleterious effects of excessive inflammation.

## 4 Materials and methods

Reagents and materials: Anti-NRF2 antibody (ab137550) was from Abcam. High-Capacity cDNA Reverse Transcription Kit (4368814) and SYBR Green PCR Master Mix (4364344) were from Applied Biosystems. Beas-2B cells (CRL-9609) were from ATCC. Soft tissue homogenizing CK14 (P000933-LYSK0-A) and Precellys - Tissue Homogenizer were from Bertin. Easy Prep RNA Miniprep Plus Kit (R01-04) was from Bioland Scientific. DC Protein Assay Reagent A/B/S (500-0113/0114/0115) was from BioRad. Anti-DCAF1 antibody (14966), Anti-HO-1 antibody (mouse specific) (86806), Anti-NRF2 antibody (12721) and K48-linkage Specific Polyubiquitin antibody (8081S) were from Cell signaling technology. Custom protein-protein interaction inhibitor drug library was from ChemDIV. CHX (BML-GR310) was from Enzo. DMEM/F-12 (11320082), Fetal Bovine Serum (26140079), Geneticin Selective Antibiotic (G418 Sulfate) (10131027), IMDM (12440061) and Opti-MEM I Reduced Serum Medium (31985062) were from Gibco. WT HAP1 cells and KEAP1-KO HAP1 cells (HZGHC003774c005) were from Horizon. Anti-β-actin antibody (MA5-15739), Countess II Automated Cell Counter (AMQAX1000), IL-1 beta Human ELISA Kit (88-7261-88), IL-1 beta Mouse ELISA Kit (88-7013-88), IL-6 Mouse ELISA Kit (88-7064-88), Mouse IgG Isotype Control (10400C), TNF alpha Human ELISA Kit (88-7346-88) and TNF alpha Mouse ELISA Kit (88-7324-88) were from Invitrogen. Amaxa Nucleofector II was from Lonza. Cell-Based Proteasome-Glo Assays (G8660, G8860), CellTiter 96 AQueous One Solution Cell Proliferation Assay (G3582), Dual-Glo Luciferase Assay System (E2920), Nano-Glo Luciferase Assay System (N1120), pGL4.37[luc2P/ARE/Hygro] Vector (E3641) and pNLF1-NRF2 [CMV/neo] Vector (N1391) were from Promega. Anti-DCAF1 antibody (mouse specific) (sc-376850), Anti-GPx-1/2 Antibody (sc-133160), Anti-HO-1 antibody (sc-136960) and Anti-NRF2 antibody (mouse specific) (sc-365949) were from Santa Cruz Biotechnology. Dimethyl fumarate (242926-25G), MISSION esiRNA library, X-tremeGENE™ HP DNA Transfection Reagent (6366244001) and X-tremeGENE siRNA Transfection Reagent (4476115001) were from Sigma-Aldrich. Protein A/G Magnetic Beads (88802) and SuperSignal West Femto Maximum Sensitivity Substrate (34095) were from Thermo Scientific. pCMV-SPORT6/VPRBP (BC110371) was from Transomic technologies. MG132 (F1100) was from UBPBio.

Cell culture: Beas-2B and MLE-12 cells from ATCC were cultured in HITES media supplemented with 10% fetal bovine serum (FBS). Wild type (WT) and KEAP1 knock-out HAP1 cells were cultured in IMDM media supplemented with 10% FBS.

Plasmid transfection: Plasmid transfections were performed in the way of nucleofection in Beas-2B and MLE-12 cells using Nucleofector II (Amaxa). X-tremeGENE HP DNA transfection reagent was used for plasmid transfections in both types of HAP1 cells.

siRNA knockdown: For the E3 ligase screening, 0.2 μg of siRNA was taken from each well of the E3 siRNA library, mixed with X-tremeGENE siRNA transfection reagent and diluted in Opti-MEM media. The mixture stood in room temperature for 20 min before 20 μl of full media holding 5 × 10^3^ cells were added. Nano-glo luciferase assays were performed after 24 h of transfection. For specific gene silencing, small interfering RNAs were selected and purchased from IDT, and transfected in cells using X-tremeGENE siRNA transfection reagent, with Negative Control DsiRNA transfected as control. Subsequent analysis was performed after 24 h of transfection.

Luminescence assays: Nano-glo luciferase assay system was used to detect signals from Beas-2B and HAP1 cells with stable overexpression of pNLF1-NRF2. Dual-luciferase reporter assay system was employed to detect signals from overexpression of pGL4.37[luc2P/ARE/Hygro]. Proteasome-glo assays were used to measure the proteasome activities. All the assay systems above were purchased from Promega and manufacturer's protocols were followed. Signals were collected and quantified using CLARIOstar plate reader from BMG Labtech.

High throughput liquid handling: Agilent Bravo automated liquid-handling platform was used to transfer contents of E3 siRNA library or Protein-protein interaction compound library into assay plates. Biotek EL406 washer dispenser was used to distribute reagents or cell solutions into assay plates. For multiple plates operation, plate and liquid handling sequence and intervals were controlled through Agilent VWORKs software.

Cell viability assessment: CellTiter 96 AQueous one solution cell proliferation assay (MTS) was used to determine cell viability changes upon drug treatment. Beas-2B cells were seeded in the 96-well cell culture plate, with 1.5 × 10^4^ cells in 75 μl full media for each well. Different concentrations of the compound BC-1901S were dissolved in 25 μl serum-free media and added. After overnight treatment, 20 μl of MTS reagent was dispensed to each well. The plate was incubated in 37°C incubator for 3 hrs, prevented from light. Absorbance signals at 490 nm were acquired by the CLARIOstar plate reader.

Western blot: Cells were lysed in RIPA buffer supplemented with EDTA-free protease inhibitor tablet on ice. Cell lysates were sonicated at 20% amplification for 12 s and centrifuged at 12,000 g for 10 min under 4°C. Supernatants were collected and normalized for the total protein concentrations, mixed with 3× protein sample buffer, and incubated at 70°C for 10 min. Samples were loaded onto the 4–12% Bis-Tris Plus Gels from Invitrogen and electrophoresed in MOPS buffer. The proteins were then electrotransferred to nitrocellulose membranes. Blots were incubated in 15 ml of blocking buffer for 1 h at room temperature, before incubation in 10 ml primary antibody solution overnight at 4°C. Three times of 10-min washing were performed in 15 ml TBST. Blots were then incubated in 10 ml secondary antibody solution for 1 h at room temperature. After three times of 10-min washing in 15 ml TBST, blots were then developed using West Femto Maximum Sensitivity Substrate from Thermo Scientific, and imaged using ChemiDoc Imaging System from Bio-Rad.

Immunoprecipitation: Cells were lysed with IP buffer (0.25% Triton X-100 in 1 × PBS supplemented with EDTA-free protease inhibitor tablet) on ice. Lysates were sonicated at 20% amplification for 12 s and centrifuged at 12,000 g for 10 min under 4°C. Supernatants were collected and normalized for the total protein concentrations. The specific antibody for pull-down was added to the supernatants with a 1:100 dilution for 2 h at 25°C. The protein-antibody complex was captured with 10 μl magnetic protein A/G beads per 1 ml lysate for 1 h. After washing with 1 ml IP buffer twice, protein was eluted in 50 mM Tris HCl pH 6.8, 2% SDS, 10% glycerol, and 100 mM DTT. After 5 min incubation at 88°C, samples were collected and used for subsequent immunoblotting.

RT-qPCR: Total RNA was extracted using RNA Extraction Miniprep Kit from Bioland Scientific, following manufacturer's manual. cDNA was prepared using High-Capacity RNA-to-cDNA Kit from Applied Biosystems. SYBR Green Real-Time PCR Master Mixes from Applied Biosystems were used in qPCR, detecting the expression level of the following genes, with primer sequences indicated: *HO-1* (sense: 5′-ATGGCCTCCCTGTACCACATC-3’; anti-sense: 5′-TGTTGCGCTCAATCTCCTCCT-3′), *GPx2* (sense: 5′-GTGCTGATTGAGAATGTGGC-3’; anti-sense: 5′-AGGATGCTCGTTCTGCCA-3′), *GAPDH* (sense: 5′-TGTCAAGCTCATTTCCTGGTAT-3’; anti-sense: 5′-CTCTCTTCCTCTTGTGCTCTTG-3′).

ELISA: Supernatants from LPS-stimulated human peripheral blood mononuclear cells (PBMCs) culture plates, with or without compound treatment, were collected to detect human TNF-α, IL-1β concentrations. A Cytokine 35-Plex Human Panel was also run for these samples. Supernatants from LPS-stimulated MLE cell culture dishes, and bronchoalveolar lavage fluid (BALF) from animal experiments were collected to detect mouse IL-6, TNF-α and IL-1β concentrations. The manufacturer's protocol was exactly followed. Absorbance readouts at the wavelengths of 450 nm and 570 nm were both acquired by the CLARIOstar plate reader.

Cellular thermal shift assay (CETSA): Beas-2B cells treated with vehicle or BC-1901S (10 μM, 1h) were lysed and used to determine DCAF1 and KEAP1 protein melting curves, following the protocol described in previous literature [31].

Mouse experiments: C57BL/6J mice were purchased from the Jackson Laboratory. Animals were between 7 and 9 weeks of age and around 25g weight at time of experiment. All procedures were approved by the University of Pittsburgh Institutional Animal Care and Use Committee. Mice were deeply anesthetized. LPS (3 mg/kg) was administered i.t. before BC-1901S (5 or 25 mg/kg, corn oil formulation) was administered to the mice through i.p. injection. Eighteen hours later, animals were euthanized, and lungs were lavaged with normal saline and harvested for subsequent analysis. Bronchoalveolar lavage fluid (BALF) was analyzed for cell count, protein assay and ELISA. Left lobes were sent for paraffin sections and H&E staining. Part of right lobes were homogenized in RIPA buffer for immunoblotting analysis.

Statistical analysis: Statistical comparisons were performed in GraphPad Prism. Unpaired two-tailed Student's *t*-test was used to compare two groups. Comparisons of more than two groups were tested with one-way ANOVA.

## Author contributions

BBC and YL designed and directed the study. YC, JWE, JRK and BBC analyzed the data, prepared the figures, and wrote the manuscript. YC, JWE, TBL, FT, JRK, YL and BBC performed all experiments and assisted with animal experiments. DPC and MSS assisted with high-throughput screening. BBC, YL and JWE provided funding for the studies.

## Declaration of competing interest

None.

## Appendix A Supplementary data

Multimedia component Figure. S1. NRF2-NanoLuc readouts of pNLF1-NRF2 overexpression Beas2B stable cells treated with vehicle or MG132 (1 μM overnight) with Z′-factor calculation. Figure S2. Top hit G856-6116 is a proteasome inhibitor. A. Structure of G856-6116. B. Immunoblotting analysis of NRF2, p21 and K48-Ub in B2B cells treated with G856-6116, DMF or MG132 of indicated concentrations. C. Chymotrypsin-like activity assay of Beas2B cells treated with G856-6116 or MG132 of indicated concentrations. Figure S3. PBMCs were treated with LPS or LPS and BC-1901S at various doses for 18h. Cell culture supernatants were then assayed for IL-8, MIP-1beta and IL-1alpha using ELISA. Data and mean ± SD of 3 independent experiments. Figure S4. Docking study (Discovery Studio 3.5) of a candidate inhibitor BC-1901S within the DCAF1 Beta-propeller domain suggests muliple interactions of protein residues with the compound. Multimedia component

Multimedia component Table S1: Normalized NRF2-NanoLuc signals of 24 top “Hit” compounds from the primary screening at gradient concentrations. Table S2: NRF2-NanoLuc signal of the Analog/derivative(s) of F869-0035, D398–0620 and T622-0510 tested in a dose course in Beas-2B cells stably expressing NRF2-NanoLuc. Table S3: Top protein hits from the esiRNA screen Multimedia component

## References

[1] Niture S.K., Khatri R., Jaiswal A.K. (2014 Jan). Regulation of Nrf2-an update. Free Radic. Biol. Med..

[2] Itoh K., Mimura J., Yamamoto M. (2010 Dec 1). Discovery of the negative regulator of Nrf2, Keap1: a historical overview. Antioxidants Redox Signal..

[3] Reddy N.M., Potteti H.R., Mariani T.J., Biswal S., Reddy S.P. (2011 Dec). Conditional deletion of Nrf2 in airway epithelium exacerbates acute lung injury and impairs the resolution of inflammation. Am. J. Respir. Cell Mol. Biol..

[4] Zhao H., Eguchi S., Alam A., Ma D. (2017 Feb 1). The role of nuclear factor-erythroid 2 related factor 2 (Nrf-2) in the protection against lung injury. Am. J. Physiol. Lung Cell Mol. Physiol..

[5] Athale J., Ulrich A., MacGarvey N.C., Bartz R.R., Welty-Wolf K.E., Suliman H.B. (2012 Oct 15). Nrf2 promotes alveolar mitochondrial biogenesis and resolution of lung injury in Staphylococcus aureus pneumonia in mice. Free Radic. Biol. Med..

[6] Wei J., Chen G., Shi X., Zhou H., Liu M., Chen Y. (2018 Jun 7). Nrf2 activation protects against intratracheal LPS induced mouse/murine acute respiratory distress syndrome by regulating macrophage polarization. Biochem. Biophys. Res. Commun..

[7] Thimmulappa R.K., Lee H., Rangasamy T., Reddy S.P., Yamamoto M., Kensler T.W. (2006 Apr). Nrf2 is a critical regulator of the innate immune response and survival during experimental sepsis. J. Clin. Invest..

[8] Cho H.-Y., Jedlicka A.E., Reddy S.P.M., Kensler T.W., Yamamoto M., Zhang L.-Y. (2002 Feb). Role of NRF2 in protection against hyperoxic lung injury in mice. Am. J. Respir. Cell Mol. Biol..

[9] Cheng X., He S., Yuan J., Miao S., Gao H., Zhang J. (2016 Apr). Lipoxin A4 attenuates LPS-induced mouse acute lung injury via Nrf2-mediated E-cadherin expression in airway epithelial cells. Free Radic. Biol. Med..

[10] Itoh K., Wakabayashi N., Katoh Y., Ishii T., Igarashi K., Engel J.D. (1999 Jan 1). Keap1 represses nuclear activation of antioxidant responsive elements by Nrf2 through binding to the amino-terminal Neh2 domain. Genes Dev..

[11] Itoh K., Wakabayashi N., Katoh Y., Ishii T., O'Connor T., Yamamoto M. (2003 Apr). Keap1 regulates both cytoplasmic-nuclear shuttling and degradation of Nrf2 in response to electrophiles. Gene Cell..

[12] Lo J.Y., Spatola B.N., Curran S.P. (2017 Apr 28). WDR23 regulates NRF2 independently of KEAP1. PLoS Genet..

[13] Wu T., Zhao F., Gao B., Tan C., Yagishita N., Nakajima T. (2014 Apr 1). Hrd1 suppresses Nrf2-mediated cellular protection during liver cirrhosis. Genes Dev..

[14] Chowdhry S., Zhang Y., McMahon M., Sutherland C., Cuadrado A., Hayes J.D. (2013 Aug 8). Nrf2 is controlled by two distinct β-TrCP recognition motifs in its Neh6 domain, one of which can be modulated by GSK-3 activity. Oncogene.

[15] Morreale F.E., Walden H. (2016 Mar 24). Types of ubiquitin ligases. Cell.

[16] Komander D., Rape M. (2012 Apr 10). The ubiquitin code. Annu. Rev. Biochem..

[17] Jin J., Xiao Y., Hu H., Zou Q., Li Y., Gao Y. (2015 Jan 7). Proinflammatory TLR signalling is regulated by a TRAF2-dependent proteolysis mechanism in macrophages. Nat. Commun..

[18] McKelvey A.C., Lear T.B., Dunn S.R., Evankovich J., Londino J.D., Bednash J.S. (2016 Nov 2). RING finger E3 ligase PPP1R11 regulates TLR2 signaling and innate immunity. Elife.

[19] Lear T., McKelvey A.C., Rajbhandari S., Dunn S.R., Coon T.A., Connelly W. (2016 May 30). Ubiquitin E3 ligase FIEL1 regulates fibrotic lung injury through SUMO-E3 ligase PIAS4. J. Exp. Med..

[20] Hu H., Wang H., Xiao Y., Jin J., Chang J.-H., Zou Q. (2016 Mar 7). Otud7b facilitates T cell activation and inflammatory responses by regulating Zap70 ubiquitination. J. Exp. Med..

[21] Chang M., Jin W., Sun S.-C. (2009 Oct). Peli1 facilitates TRIF-dependent Toll-like receptor signaling and proinflammatory cytokine production. Nat. Immunol..

[22] Coon T.A., McKelvey A.C., Lear T., Rajbhandari S., Dunn S.R., Connelly W. (2015 Jul 8). The proinflammatory role of HECTD2 in innate immunity and experimental lung injury. Sci. Transl. Med..

[23] Marzec J.M., Christie J.D., Reddy S.P., Jedlicka A.E., Vuong H., Lanken P.N. (2007 Jul). Functional polymorphisms in the transcription factor NRF2 in humans increase the risk of acute lung injury. Faseb. J..

[24] Abed D.A., Goldstein M., Albanyan H., Jin H., Hu L. (2015 Jul 2). Discovery of direct inhibitors of Keap1-Nrf2 protein-protein interaction as potential therapeutic and preventive agents. Acta Pharm. Sin. B.

[25] Robledinos-Antón N., Fernández-Ginés R., Manda G., Cuadrado A. (2019 Jul 14). Activators and inhibitors of NRF2: a review of their potential for clinical development. Oxid. Med. Cell Longev..

[26] Hast B.E., Goldfarb D., Mulvaney K.M., Hast M.A., Siesser P.F., Yan F. (2013 Apr 1). Proteomic analysis of ubiquitin ligase KEAP1 reveals associated proteins that inhibit NRF2 ubiquitination. Canc. Res..

[27] Kobayashi E.H., Suzuki T., Funayama R., Nagashima T., Hayashi M., Sekine H. (2016 May 23). Nrf2 suppresses macrophage inflammatory response by blocking proinflammatory cytokine transcription. Nat. Commun..

[28] Battino M., Giampieri F., Pistollato F., Sureda A., de Oliveira M.R., Pittalà V. (2018). Nrf2 as regulator of innate immunity: a molecular Swiss army knife!. Biotechnol. Adv..

[29] Chan K., Kan Y.W. (1999 Oct 26). Nrf2 is essential for protection against acute pulmonary injury in mice. Proc. Natl. Acad. Sci. U.S.A..

[30] Rada P., Rojo A.I., Chowdhry S., McMahon M., Hayes J.D., Cuadrado A. (2011 Mar). SCF/{beta}-TrCP promotes glycogen synthase kinase 3-dependent degradation of the Nrf2 transcription factor in a Keap1-independent manner. Mol. Cell Biol..

[31] Jafari R., Almqvist H., Axelsson H., Ignatushchenko M., Lundbäck T., Nordlund P. (2014 Sep). The cellular thermal shift assay for evaluating drug target interactions in cells. Nat. Protoc..

[32] Jiang Z.-Y., Lu M.-C., Xu L.L., Yang T.-T., Xi M.-Y., Xu X.-L. (2014 Mar 27). Discovery of potent Keap1-Nrf2 protein-protein interaction inhibitor based on molecular binding determinants analysis. J. Med. Chem..

[33] Marcotte D., Zeng W., Hus J.-C., McKenzie A., Hession C., Jin P. (2013 Jul 15). Small molecules inhibit the interaction of Nrf2 and the Keap1 Kelch domain through a non-covalent mechanism. Bioorg. Med. Chem..

[34] Kansanen E., Kuosmanen S.M., Leinonen H., Levonen A.-L. (2013 Jan 18). The Keap1-Nrf2 pathway: mechanisms of activation and dysregulation in cancer. Redox Biol..

[35] Lau A., Villeneuve N.F., Sun Z., Wong P.K., Zhang D.D. (2008 Dec). Dual roles of Nrf2 in cancer. Pharmacol. Res..

