# Supplementary figures and images for: A small molecule NRF2 activator BC-1901S ameliorates inflammation through DCAF1/NRF2 axis
Source: Redox Biol. 2020 Mar 4;32:101485. doi: 10.1016/j.redox.2020.101485 (PMC7068124; doi:10.1016/j.redox.2020.101485)

Figure S1

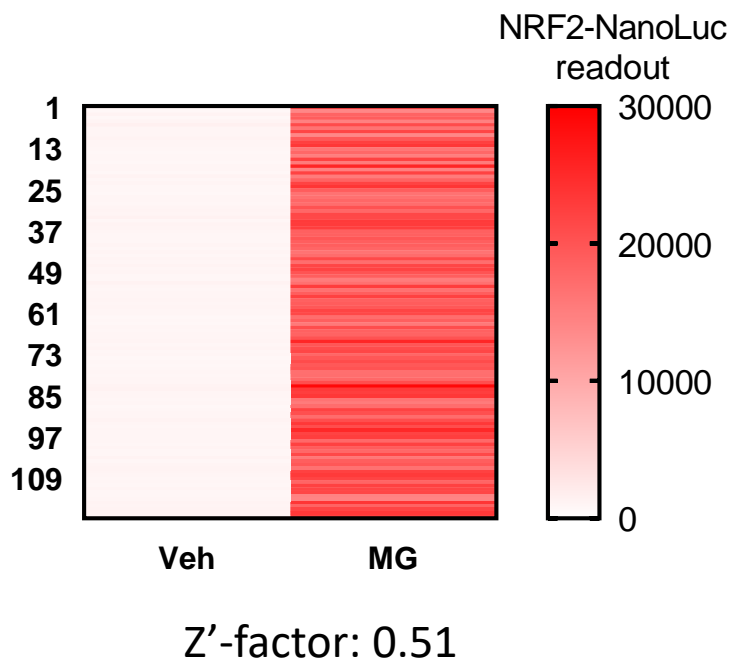

Figure S2

A

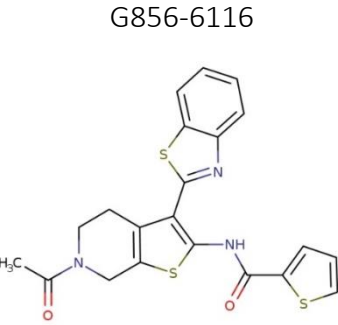

B

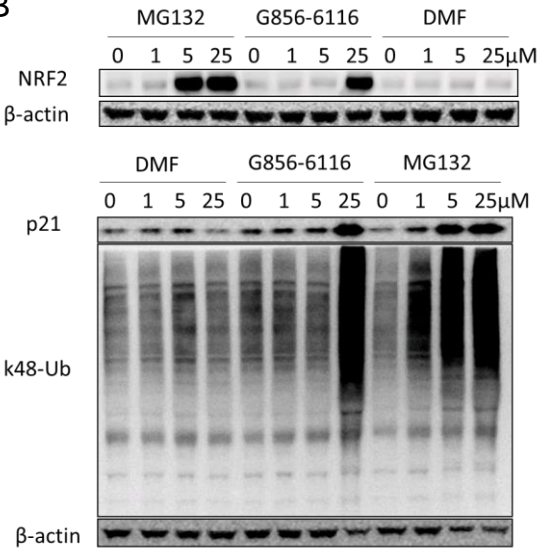

C

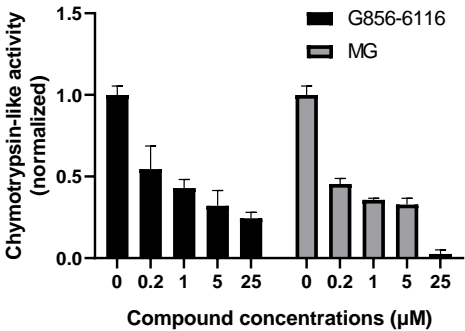

Figure S3

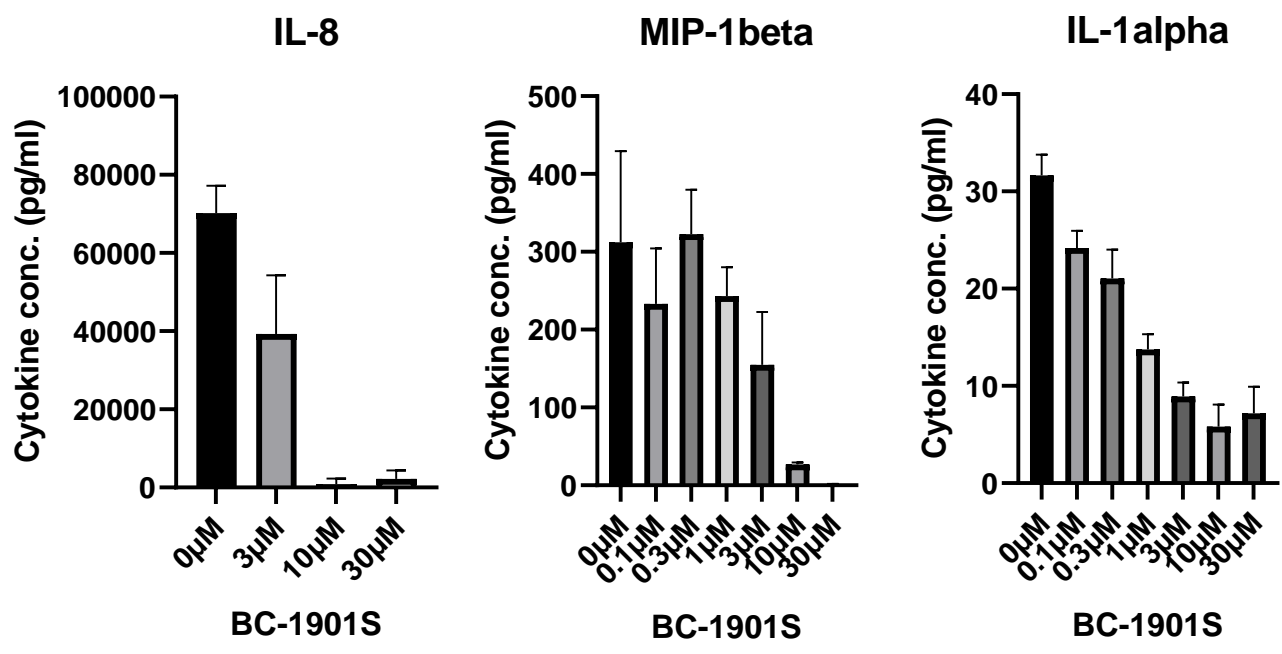

Figure S4

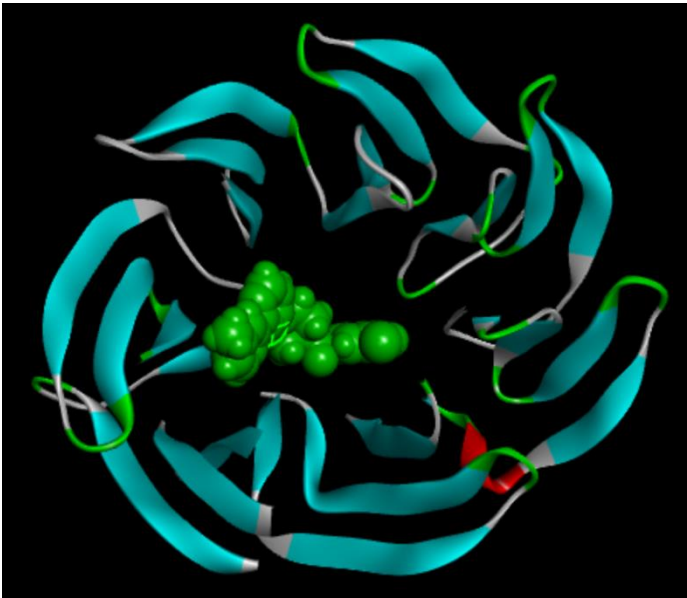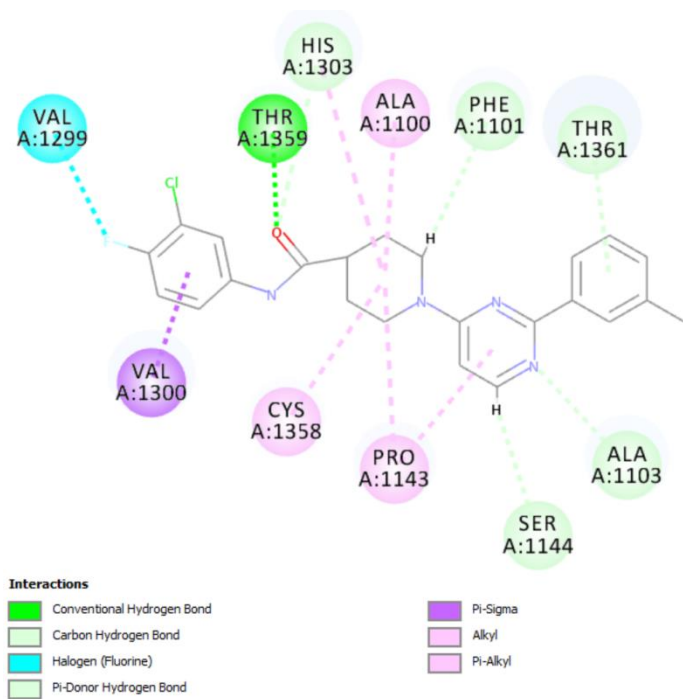

Supplement: Multimedia component — Figure. S1. NRF2-NanoLuc readouts of pNLF1-NRF2 overexpression Beas2B stable cells treated with vehicle or MG132 (1 μM overnight) with Z′-factor calculation. Figure S2. Top hit G856-6116 is a proteasome inhibitor. A. Structure of G856-6116. B. Immunoblotting analysis of NRF2, p21 and K48-Ub in B2B cells treated with G856-6116, DMF or MG132 of indicated concentrations. C. Chymotrypsin-like activity assay of Beas2B cells treated with G856-6116 or MG132 of indicated concentrations. Figure S3. PBMCs were treated with LPS or LPS and BC-1901S at various doses for 18h. Cell culture supernatants were then assayed for IL-8, MIP-1beta and IL-1alpha using ELISA. Data and mean ± SD of 3 independent experiments. Figure S4. Docking study (Discovery Studio 3.5) of a candidate inhibitor BC-1901S within the DCAF1 Beta-propeller domain suggests muliple interactions of protein residues with the compound. [file mmc1.pdf]
