# Supplementary material for: A small molecule NRF2 activator BC-1901S ameliorates inflammation through DCAF1/NRF2 axis
Source: Redox Biol. 2020 Mar 4;32:101485. doi: 10.1016/j.redox.2020.101485 (PMC7068124; doi:10.1016/j.redox.2020.101485)
Supplement: Multimedia component — Table S1: Normalized NRF2-NanoLuc signals of 24 top “Hit” compounds from the primary screening at gradient concentrations. Table S2: NRF2-NanoLuc signal of the Analog/derivative(s) of F869-0035, D398–0620 and T622-0510 tested in a dose course in Beas-2B cells stably expressing NRF2-NanoLuc. Table S3: Top protein hits from the esiRNA screen [file mmc2.pdf]

TABLE S1

	T622-0510	T622-0158	3568-0108	Y020-1093	T622-0548	T815-1119	T965-0869	T479-0307	T633-2591	D398-0620	E470-0488	E538-0203	D715-0442	D715-0530	E859-0056	E786-0108	F869-0035	G216-0296	G856-6116	J005-0540	M475-0143	1418-0044	T993-0873	5988-0062
0	0.977	0.929	0.896	0.900	1.106	1.161	3.932	1.112	0.843	0.947	0.885	0.835	1.172	0.953	1.140	0.946	1.057	1.017	0.925	0.945	1.050	0.952	1.168	1.084
0.0016	0.951	0.946	0.915	1.102	0.913	1.245	1.107	0.923	0.820	0.950	0.912	0.915	0.946	1.072	0.998	1.018	1.084	0.943	1.022	1.036	0.965	1.025	1.157	1.145
0.008	0.968	0.821	1.000	0.854	0.964	0.915	0.885	0.898	0.925	0.868	0.907	1.008	1.015	0.991	0.929	0.942	0.801	0.935	0.775	0.827	0.906	0.911	1.058	0.942
0.04	1.067	0.832	0.995	1.019	1.132	0.966	0.917	0.880	0.915	0.821	0.810	0.920	1.214	0.915	0.855	0.939	0.792	0.923	1.166	0.890	0.895	0.825	1.051	1.004
0.2	1.046	0.877	0.838	0.957	0.932	0.850	0.806	0.858	0.953	0.866	0.930	1.050	1.033	0.981	0.907	0.858	0.917	0.784	0.945	1.029	0.809	1.178	0.943	0.931
1	1.050	1.069	0.939	0.903	0.879	1.032	1.003	0.902	0.921	1.017	0.917	0.949	1.283	0.952	0.915	1.015	0.916	0.903	1.009	0.905	0.841	0.841	0.943	0.957
5	1.209	1.005	0.953	0.832	1.051	0.917	1.026	0.924	0.938	1.074	0.900	1.051	0.929	1.004	0.876	0.996	1.353	1.118	4.816	1.019	0.987	0.917	0.883	1.007
25	1.546	1.209	1.058	0.972	1.076	0.983	1.098	1.019	1.200	1.639	1.039	1.414	1.070	1.010	0.986	1.340	12.370	2.619	123.495	2.248	0.946	0.998	1.030	1.058

TABLE S2

A

	25µM	5µM	1µM	0.2µM
D398-0063	1.506	1.018	0.977	1.040
S646-0245	1.434	1.103	0.968	1.023
D398-0347	1.425	1.116	0.981	0.936
D398-0646	1.419	1.063	0.991	0.951
D398-0620	1.399	1.088	0.960	0.930
D398-1117	1.365	1.007	1.000	1.054
T829-1947	1.362	1.023	0.930	0.925
D398-0634	1.358	1.080	0.929	0.988
D398-0538	1.347	1.110	0.987	0.948
D398-0156	1.340	1.022	1.033	0.984
D398-0253	1.340	1.019	0.885	0.962
D398-0621	1.331	1.221	1.101	1.025
D398-0522	1.323	0.979	1.004	1.006
D398-0064	1.296	1.067	0.891	0.970
D398-0444	1.293	1.192	0.961	0.977
T829-1968	1.288	1.013	0.962	0.988
Y200-4546	1.287	1.076	0.955	1.030
D398-0618	1.282	1.018	0.993	1.007
D398-0539	1.267	1.048	1.029	0.987
S561-0501	1.262	0.985	0.937	0.971
D398-0061	1.260	1.059	0.999	1.015
SA44-0312	1.253	1.077	1.122	0.936
D398-0060	1.241	1.108	1.005	0.955
L239-0298	1.237	1.027	0.937	0.946
D398-0251	1.233	0.995	0.947	0.971
D398-0637	1.231	1.134	0.955	0.944
D398-0613	1.227	1.014	0.937	1.000
D398-0332	1.221	1.077	1.008	0.965
D398-0645	1.219	0.980	0.952	0.936
D398-0046	1.212	1.078	0.986	0.947
D398-0252	1.200	1.117	0.907	0.953
D398-0523	1.193	1.062	1.007	1.063
D398-0638	1.178	1.016	0.974	1.108
D398-0349	1.165	0.953	1.040	0.989
D398-1299	1.159	1.041	0.984	1.038
D398-0071	1.159	1.000	0.971	0.966
D398-0348	1.152	1.045	0.976	0.999
D398-0047	1.142	0.974	0.983	1.049
D398-0540	1.135	1.063	0.982	1.066
D398-0633	1.109	1.087	1.037	0.928
D398-0039	1.079	1.033	1.003	0.984
D398-0236	1.044	0.978	0.953	1.129
D398-0072	1.038	1.019	1.025	1.085

B

	25µM	5µM	1µM	0.2µM
SA59-0041	1.446	1.214	0.965	0.942
T622-0506	1.413	1.088	1.009	0.922
S672-0564	1.347	1.078	1.064	1.040
T622-0150	1.334	1.066	0.995	0.893
S223-0293	1.331	1.042	0.885	0.906
S232-0396	1.330	1.056	0.937	0.928
L629-0054	1.328	1.111	1.080	1.057
S329-4213	1.326	1.001	0.897	1.068
T622-0146	1.317	0.996	0.916	0.955
S635-3355	1.307	1.015	1.051	0.938
L629-0483	1.301	1.203	1.089	1.048
S213-0510	1.301	1.062	0.928	0.932
S511-0940	1.271	1.105	0.940	0.902
S910-0041	1.268	1.039	1.076	1.055
S635-4216	1.261	1.097	0.937	1.002
S511-1798	1.256	1.027	0.955	0.912
S511-1226	1.255	0.991	0.951	0.968
S649-1027	1.248	1.169	0.966	0.953
S662-0248	1.226	1.103	0.979	1.017
T622-0510	1.212	1.018	0.992	0.930
S635-3812	1.202	1.093	1.023	1.110
CM4575-1890	1.196	1.032	0.911	0.952
S511-1512	1.161	1.010	0.942	0.982
S726-0860	1.157	1.031	0.961	0.913
S890-0049	1.150	1.016	0.917	1.053
S511-2084	1.146	1.072	0.927	0.940
T500-1525	1.131	1.042	0.956	0.965
S997-1225	1.114	0.963	0.888	0.956
M574-0652	1.103	1.004	1.004	0.971
S330-4119	1.094	1.073	0.967	1.031
S726-1438	1.046	1.090	0.895	0.964

C

	25µM	5µM	1µM	0.2µM
F869-0056	29.471	1.842	1.066	1.010
F869-0035	9.423	1.164	1.101	1.065
F869-0673	7.280	0.716	0.647	0.766
F869-0698	7.244	0.916	1.159	0.999
F869-0659	6.529	0.917	0.899	0.956
F726-0656	2.939	0.854	1.125	1.077
F869-0031	2.526	0.978	1.074	0.921
F869-0647	2.342	0.805	0.809	0.892
F869-0666	2.052	0.900	0.944	0.854
F869-0367	1.943	0.800	0.923	0.950
F869-0348	1.895	0.951	1.012	0.903
F869-0686	1.745	0.813	0.997	0.932
F869-0694	1.627	0.764	0.833	0.850
F869-0716	1.570	0.781	0.854	0.932
F869-0682	1.496	0.876	0.836	0.995
F869-0036	1.475	0.800	0.951	1.013
F869-0691	1.468	0.889	0.950	1.253
F869-0654	1.384	0.842	0.932	0.854
F869-0656	1.260	0.784	0.838	0.943
F869-1004	1.179	0.835	0.932	0.911
F869-0352	1.146	0.903	0.973	0.934
F869-0723	1.141	0.757	0.892	0.938
F869-0641	1.141	0.972	0.921	0.946
F869-0086	1.138	0.929	0.926	0.944
F869-0660	1.137	0.875	0.881	0.949
F869-1003	1.067	0.853	0.882	0.961
F869-0662	1.057	0.824	0.866	0.899
F869-0679	1.048	0.906	0.879	0.900
F869-0667	1.036	0.810	0.803	1.084
F869-0710	1.030	0.763	0.909	0.879
F726-0667	1.023	1.116	1.057	1.066
F869-0669	1.020	0.823	0.856	0.914
F869-0042	1.013	0.922	1.018	1.070
F869-0061	1.010	0.965	0.962	1.007
F869-0350	1.001	0.932	0.988	0.963
F869-0068	0.989	0.944	1.007	0.973
F869-0693	0.973	0.852	0.896	0.960
F869-1009	0.965	0.839	1.056	0.908
F726-0654	0.964	0.896	1.082	0.917
F869-0093	0.921	0.833	0.960	0.998
F869-0981	0.906	0.860	0.863	0.869
F869-1294	0.901	0.854	0.958	0.982
F869-0019	0.900	0.962	1.138	0.962
F869-1290	0.899	0.970	0.958	0.883
F869-1303	0.896	0.730	0.833	0.887
F869-0718	0.887	0.761	0.942	0.906
F869-0005	0.886	0.934	0.999	1.040
F726-0685	0.872	0.926	1.041	0.983
F869-0950	0.867	0.982	0.971	0.913
F869-0658	0.862	0.779	0.785	0.887
F869-0370	0.861	0.842	0.906	0.908
F869-0344	0.835	0.778	0.876	1.005
F869-0349	0.833	0.962	0.980	1.005
F869-0357	0.816	0.935	0.983	0.952
F869-0049	0.808	0.904	1.020	0.976
F869-1292	0.804	0.992	1.018	1.017
F869-0041	0.796	0.916	1.023	0.960
F869-1284	0.796	0.936	0.878	0.901
F869-0360	0.782	0.875	0.927	0.900
F869-0032	0.775	0.943	1.082	0.989
F869-0632	0.773	0.927	0.949	0.987
F869-0997	0.757	0.887	1.004	0.884
F869-0063	0.750	0.892	1.019	0.993
F869-0671	0.746	0.692	0.851	0.966
F869-0067	0.731	0.872	1.022	0.929
F869-0084	0.727	0.777	0.995	0.955
F869-0697	0.718	0.801	0.782	0.848
F869-0649	0.710	0.911	1.011	0.907
F869-0064	0.697	0.835	0.930	0.898
F869-0024	0.684	0.771	0.968	1.028
F869-0039	0.669	0.895	0.992	0.925
F869-0644	0.664	0.871	0.929	1.002
F869-0070	0.658	0.892	0.944	0.936
F869-0724	0.643	0.792	0.894	0.984
F869-0661	0.612	0.998	0.892	0.894
F869-0037	0.608	0.862	1.015	0.974
F869-0726	0.587	0.835	0.828	0.905
F869-0054	0.587	0.850	1.015	0.942
F869-0714	0.584	0.755	0.787	0.967
F869-0681	0.575	0.812	0.954	0.943
F869-0692	0.572	0.779	0.774	0.836
F869-0051	0.523	0.841	0.936	0.980

TABLE S3

Gene	Fold	Gene	Fold	Gene	Fold	Gene	Fold
UBC	53.8	PSMD14	16.0	PSMA5	8.4	CHD3	5.1
UBB	39.9	PSMD12	14.8	PSMA1	8.1	DCAF1	4.4
PSMA4	31.3	PSMD7	14.5	PSMA7	6.5	OTUD3	4.3
PSMA3	30.5	PSMB7	13.7	PSMC5	6.3	PCGF2	4.2
PSMA6	29.5	PSMB6	12.9	PSMC6	6.2	CHFR	4.1
PSMD8	26.7	PSMB4	12.5	PSMD2	5.8	MARCH11	4.1
PSMD1	25.2	PSMB1	12.3	JOSD1	5.7	KLHL6	4.0
PSMD11	23.5	PSMB5	11.7	BTBD8	5.6	PSMC4	4.0
PSMD6	20.4	POMP	11.2	PSMA2	5.5	RPP21	3.9
PSMB3	18.2	PSMD3	8.8	COPS2	5.1	HERC4	3.9
